# Biochemical and haematological assessment of toxic effects of the leaf ethanol extract of *Petroselinum crispum* (Mill) Nyman ex A.W. Hill (Parsley) in rats

**DOI:** 10.1186/1472-6882-13-75

**Published:** 2013-04-04

**Authors:** Emmanuel Olorunju Awe, S Olatunbosun Banjoko

**Affiliations:** 1 Department of Pharmacology & Therapeutics, College of Health Sciences, Ladoke,Akintola University of Technology, Osogbo campus, P.M.B 4000, Ogbomoso, Nigeria; 2 Department of Chemical Pathology, College of Health Sciences, Obafemi Awolowo University, Ile-Ife, Nigeria; 3 Institute of Public Health, College of Health Sciences, Obafemi Awolowo University, Ile-Ife, Nigeria

**Keywords:** Ethanol extract, *Petroselinum crispum* (Parsley), Toxicity, Haematology and biochemistry, Histopathology, Rats

## Abstract

**Background:**

*Petroselinum crispum,* a bright green biennial shrub is widely used traditionally as a food additive and herbal remedies for many ailments. This study therefore aimed to assess the toxic effects of its leaf extract using some biochemical, haematological parameters.

**Methods:**

The toxic effects were assessed by quantifying liver enzymes such as serum aspartate amino transferase (AST), alanine amino transferase (ALT), alkaline phosphatase (ALP), total serum protein and liver weight. Effects on haematological parameters were assessed by analysis of parked cell volume (PCV), red blood cell count (RBC), white blood cells (WBC) and haemoglobin (Hb) concentrations. Histopathological studies were done on the liver and kidneys.

**Results:**

The extract caused significant increase in serum activity of alanine amino transferase and blood urea nitrogen (BUN) levels at the dose of 1000 mg/kg. Other biochemical and haematological parameters were not affected at lower doses. Conversely, the liver weight was not affected after eight weeks of treatment at the dose levels studied. The organs obtained for pathological study, were structurally unchanged under histopathological evaluation at lower doses but inflammatory and necrotic features were observed at doses ≥ 1000 mg/kg.

**Conclusion:**

The results indicate that the leaf ethanol extract of *Petroselinum crispum* was hepatotoxic and nephrotoxic at continued oral doses equal to or more than 1000 mg/kg, but no obvious toxicity when used at lower doses. Therefore, there should be caution in its administration to avoid overdosing and known interaction with some medications. In addition, the plant should be kept away from pets and domestic animals and should not be cultivated on soil irrigated with waste water due to their ability to bio-accumulate toxic metals.

## Background

*Peteroselinum crispsum* (Parsley) is a bright green, biennial herb, which belongs to the family Apiaceae. Native to the central Mediterranean region (Southern Italy, Algeria and Tunisia) and naturalised elsewhere in Europe, Africa and Asia. It is commonly used as a ganish in soups, salads, meats, vegetables and sauces [1]. Traditionally, the leaf, seed and root are being used in herbal medicine as enema, orally as tea to control high blood pressure and tonic to strengthen the bladder [2,3]. Other ayurvedic uses of the plant includes treatment of nose bleeding, hematoma, skin blemishes due to it’s bleaching properties, halitosis, ear ache, otitis, and as an emenagogue favouring menstruation and alleviating it’s pains. The myristicin and apiole contained in the plant have the properties to increase the production of oestrogen which make their use relevant in menopause. However, large amount can have uterotonic effect and therefore it’s use is contraindicated in pregnancy and ingestion of more than 10 drops a day of the oil may cause abortion. Parsley is also widely used as a galactofuge by lactating mothers to stop excessive milk production [4,5].

Although, the use of the plant is discouraged in heart and kidney disorders due to its water retention capabilities, however, its anti inflammatory and probable immune boosting properties make it relevant in the traditional treatment of urinary tract infection, nephritis, cystitis and prevention of renal stones formation. In addition, it is also a common home remedy for obesity and reduction of itching in insect bites [5]. *Peteroselinum crispsum* increases diuresis by inhibiting the Na^+^/K^+^ - ATPase pump in the kidney, thereby enhancing sodium and water excretion while increasing potassium resorption [6,7].

With reference to herb-drug interactions, Parsley has been known to interfere with warfarin (Coumadin) treatment due to it’s high content of vitamin K [8]. It’s use is also contraindicated in diuretic treatment with potential of causing excessive loss of water. In addition, patients on aspirin therapy should avoid ingesting Parsley due to possible increased sensitivity and allergic reactions [4,8]. The presence of myristicine, a narcotic which affects the central nervous system can interfere with opoid therapy and when ingested in excess may cause convulsions and serotonin syndrome [9,10].

It is pertinent to note that the ingestion of the plant is toxic to many domestic animals including horses, cats and dogs due to the action of furocoumarins causing symptoms such as photosentisization, ulcerative and exudative dermatitis and ocular toxicity that may require veterinary consultation [11].

Consideration should also be giving to sources of plant cultivation due to the fact that resorption of heavy metals by the plants has been observed in soil irrigated with untreated waste water making the plant a potential source of heavy metal toxicity [12].

Phytochemically, the leaves and seeds of *P. crispum* has been shown to contain high levels of essential oil known as apiole, while the tender buds contain psoralen and related compounds that can induce photosensitivity and these include xanthotoxin, ficusin, bergapten, majudin, heraclin and antimicrobial furocoumarins namely 8-methoxypsoralen, 5-methoxypsoralen, oxypuecedanin, isopimpinellin, 6’-acetylopin and a new monoterpene glycoside [13-15]. The seed also contains cafiolene, beta-phelandrene, myrcene, fat and myristicin. Furthermore, the plant is a good source of iron, calcium, phosphorous and antioxidants like luteolin, vitamin C, vitamin A and zinc and these may likely account for it’s hepato protective effect [16,17]. However, in one study, combined treatment of rat with CCl_4_ and *P. crispum* extract demonstrated both synergistic and antagonistic effects on the liver [18].

While phytochemical and pharmacological experiments with *P. crispum* extract have been undertaken with disparity on its hepatotoxic effect, toxicity testing using biochemical and haematological data are not available. This present study evaluated the toxic effects of the extract of *P.crispum* on two target organs; liver and kidney, some plasma biochemical and haematological parameters.

## Methods

### Animals

The experimental protocol and procedures used in this study were approved by the Animal Ethics Committee of Ladoke Akintola University of Technology Osogbo Campus, Osun State, Nigeria; and conform to the *Guide of Care and Use of Animals in Research and Teaching* (published by the Animal Ethics Committee of Ladoke Akintola University of Technology Osogbo Campus, Osun State, Nigeria). Twenty-four wistar albino rats (220–250 g) of both sexes, divided into four groups of six animals in each group were used for the experiment. All animals were maintained on standard pellet diet (Ladokun Feeds, Nigeria limited) and water *ad libitum.*

### Plant materials

*Petroselinum crispum* (Parsley) fresh leaves were collected at Osogbo in the southern part of Nigeria. The leaves were identified by a taxonomist at the herbarium of the Federal Institute of Forest Research Ibadan where voucher specimen was deposited with Voucher Number 126698.

### Preparation of plant extract

*Petroselinum crispum* (Parsley) fresh leaves (1 kg) were air-dried at room temperature. The air-dried leaves of the plant were milled into fine powder in a Waring commercial blender. The powdered leaves were macerated in 500 ml ethanol at room temperature (27 ± 1 C) and extracted for 48 hours with occasional shaking. The soluble ethanol extract was filtered and concentrated to dryness under reduced pressure at 60 ± 1°C in rotary evaporator. Freeze-drying and solvent elimination of the resulting ethanol extract was weighed and finally gave 40.35 g. The percentage yield was calculated using this formula: weight of extract/original weight × 100 giving4.0.35% yields) of brown powdery crude *Petroselinum crispum* leaf ethanol extract (PCE). The plant extract was stored in the refrigerator and aliquot of the concentrations were prepared immediately before use and was found to be stable throughout the duration of the experiment.

### Administration of the extract

Separate groups of animals were given oral doses of the extract at 10, 100 and 1000 mg/kg body weight daily for 8 weeks. A control group was treated daily with 2 ml of the 5% Tween 80 in saline.

### Parameters

Clinical signs and death were observed daily, while weekly body weight was recorded for each animal. Blood samples were obtained after eight weeks of oral administration of the under light anaesthesia from the orbital sinus using heparinised microhaematocrit tubes according to the method of [19] and modified by [20]. Plasma was separated by centrifugation of blood samples at 3000 rpm for 5 minutes [21]. The plasma thus obtained was used in the determination of biochemical parameters were measured using Randox Laboratories, UK reagent kits.

The activity of aspartate amino transferase (AST) and alanine amino transferase (ALT) were determined colorimetrically at 546 nm using a standard method [22]. Alkaline phosphatase (AP) was measured using colometric method described by [23]. Total plasma protein (TP) values were measured colorimetrically at 546 nm using the biuret method [24]. Plasma albumin (ALB).concentration was measured colorimetrically at 630 nm using bromocresol green method [25]. The concentration of blood urea nitrogen (BUN) was determined colorimetrically at 546 nm using the urease cleavage Berthelot’s reaction described by [26].

Orbital sinus blood sample was obtained weekly in EDTA for haematological examination. The parameters measured were hemoglobin Concentration (Hb), packed cell volume (PCV), red blood cells (RBCs), platelets count white blood cells (WBCs), differential wbcs counts and erythrocytes indices; mean corpuscular volume (MCV), using automated Sysmex KX-21 haematology analyzer.

All animals were sacrificed by exsanguinations after light ether inhalation anaesthesia at the end of the experiment. The animals were subjected to post-mortem examination with collection of samples from the liver, heart, kidneys spleen pancreas, lymph nodes, stomach, intestines, Payer's patches, urinary bladder and lungs. The tissues were fixed in 10% neutral buffered formalin for at least 48 h. 4–5 μm paraffin sections were processed and stained with haematoxylin and eosin for microscopic examination using established protocols [27].

### Data analysis

Data were expressed as mean ± SEM. Statistical analyses was performed by one-way, ANOVA followed by Dunnett’s test. P values < 0.05 were considered significant.

## Results

There were no abnormal signs of toxicity or death recorded after the eight weeks of treatment at the dose range of 10-1000 mg/kg body weight. Body weight gradually increased at these doses comparable with that of control (Table 1).

**Table 1:** **Effects of oral treatment of varying doses of ethanol from *P*etroselinum crispum on biochemical parameters in rats**

**Treatment**	**Dose mg/kg**	**Liver weight(g)**	**Body weight(g)**	**TP (g/dl)**	**ALB (g/dl)**	**AST (IU/L)**	**ALT (IU/L)**	**ALP (IU/L)**	**BUN (mg/dl)**
control	-	1.36 ± 0.1	235 ± 4.0	8.0 ± 0.1	4.0 ± 0.1	44.0 ± 1.0	77 ± 2.0	77.0 ± 2.0	16.0 ± 1.0
PSE	10	1.14 ± 0.1	235 ± 4.0	7.9 ± 0.1	4.1 ± 0.1	44.0 ± 1.0	77 ± 2.0	78.0 ± 2.0	17.0 ± 1.0
PSE	100	1.11 ± 0.1	234 ± 4.0	T.8 ± 0.1	3.9 ± 0.1	45.0 ± 1.0	78 ± 2.0	77.0 ± 2.0	18.0 ± 1.0
PSE	1000	1.16 ± 0.1	235 ± 4.0	7.9 ± 0.1	3.8 ± 0.1	45.0 ± 1.0	88 ± 2.0	89.0 ± 2.0*	19.0 ± 1.0*.

*P < 0.05 when compared with control, TP = Total protein, ALB = Albumin, AST = Aspartate amino-transferase,

ALT = Alanine amino- transferase, AP = Alkaline phosphate, BUN = Blood Urea Nitrogen.

The activity of aspartate amino transferase (AST), showed no significant change in all dose levels studied. At doses of 10 or 100 mg/kg body weight compared to the control, there was also no significant change in the activity of ALT. However, a significant increase was observed at the dose of 1000 mg/kg (Table 1). The activity of plasma ALP increased at doses of 100 and 1000 mg/kg, but no significant difference was observed at lower doses of 10 mg/kg and 100 mg/kg.

BUN level at the dose of 1000 mg/kg was significantly higher than in the control; however there was no significant difference at the doses of 10 and 1000 mg/kg compared to the control. There was no significant difference in the values of TP and ALB at all dose levels studied (Table 1).

There were no significant differences in the erythrocytic parameters. PCV was not affected by all the dose levels studied. There was also no significant difference in haemoglobin concentration, RBC and MCV at all dose levels. There was however significant differences in WBC values at doses of 100 and 1000 mg/kg compared to that of 10 mg/kg and control. In the differential leukocyte count, neutrophils showed significant difference at the dose levels of 100 and 1000 mg/kg compared to the values at the dose of 10 mg/kg and control, while lymphocyte, monocyte and eosinophil counts showed no significant difference at all dose levels compared to control (Table 2).

**Table 2:** Effects of orals treatment with varying doses of ethanol extract from Petroselinium crispum on haematological biochemical parameters in rats

**Treatment**	**Dose mg/kg**	**PCV (%)**	**Hb (g/dl)**	**RBC X10**^**6**^**/μL**	**WBC X10**^**3**^**/μL**	**MCV (fl)**	**N (%)**	**M (%)**	**L (%)**	**E (%)**
Control	-	47.7 ± 4	15.5 ± 0.2	6.3 ± 0.1	15.2 ± 0.5	60.0 ± 0.4	18.6 ± 1	80.7 ± 1.0	0.3 ± 0.1	0.4 ± 1.0
PCE	10	47.8 ± 4	15.6 ± 0.2	6.2 ± 0.1	15.5 ± 0.5	60.6 ± 0.4	18.7 ± 1	80.7 ± 0.9	0.3 ± 0.1	0.3 ± 0.8
PCE	100	48.3 ± 4	16.0 ± 0.2	6.1 ± 0.1	16.5 ± 0.5	60.1 ± 0.4	18.1 ± 1	80.6 ± 0.9	0.4 ± 0.13*	0.3 ± 0.8
PCE	1000	47.7 ± 4	16.0 ± 0.2	6.3 ± 0.1	17.5 ± 0.5	60.0 ± 0.4	18.0 ± 1	82.1 ± 1.0	0.5 ± 0.17*	0.3 ± 0.8

*P < 0.05, compared to control, PCV = Packed cell Volume, Hb = Haemoglobin, RBC = Red blood cell, MCV = Mean corpuscular volume, WBC = White blood cell, N = Neutrophils, L = Lymphocyte, M = Monocyte, E = Eosinophils.

**Figure 1:**
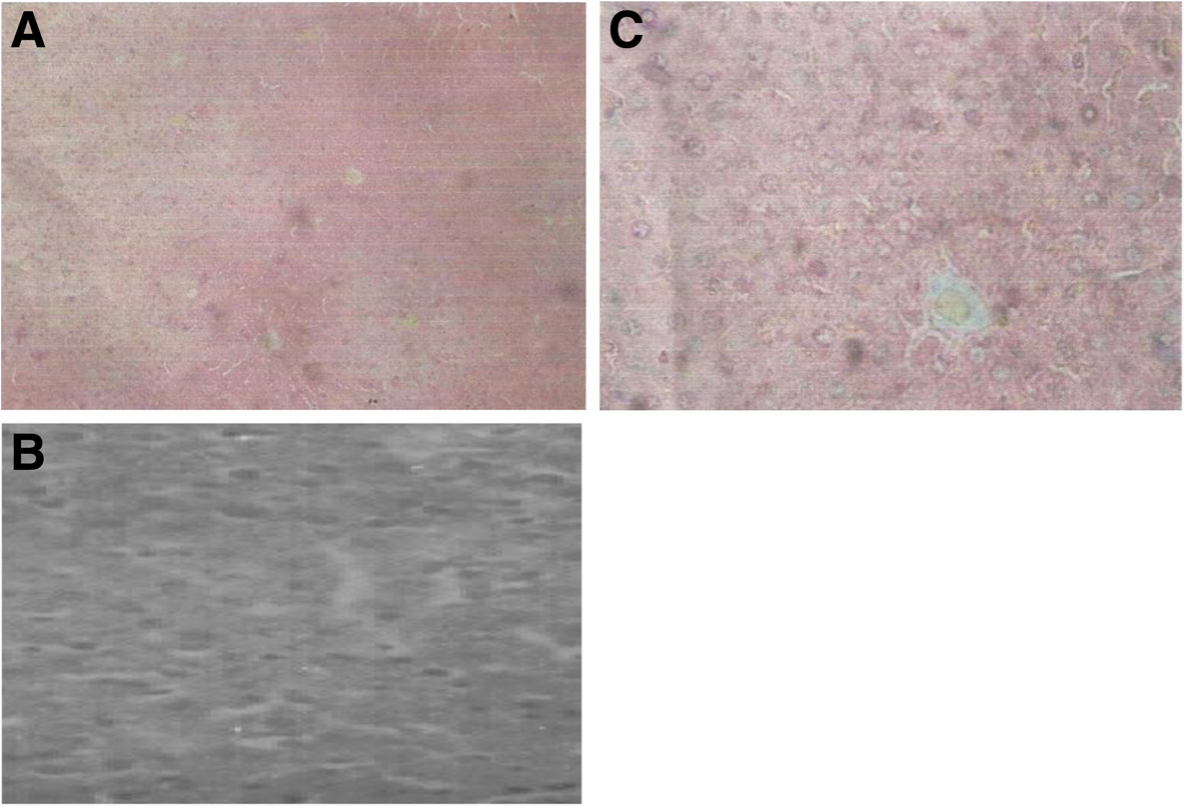
**A: Histological section of liver of the control rat (H&E x100).** Section showing normal hepatocytes. **B**: Histological section of test rat liver at a dose of 100 mg/kg (H&E x100). Showing moderate to normal hepatocytes in extract- treated animals at 100 mg/kg. **C**: Histological section of test rat liver at a dose of 1000 mg/kg (H&Ex100). Showing severe degeneration, necrosis and mild to moderate inflammatory reaction in extract treated animals at 1000 mg/kg.

**Figure 2:**
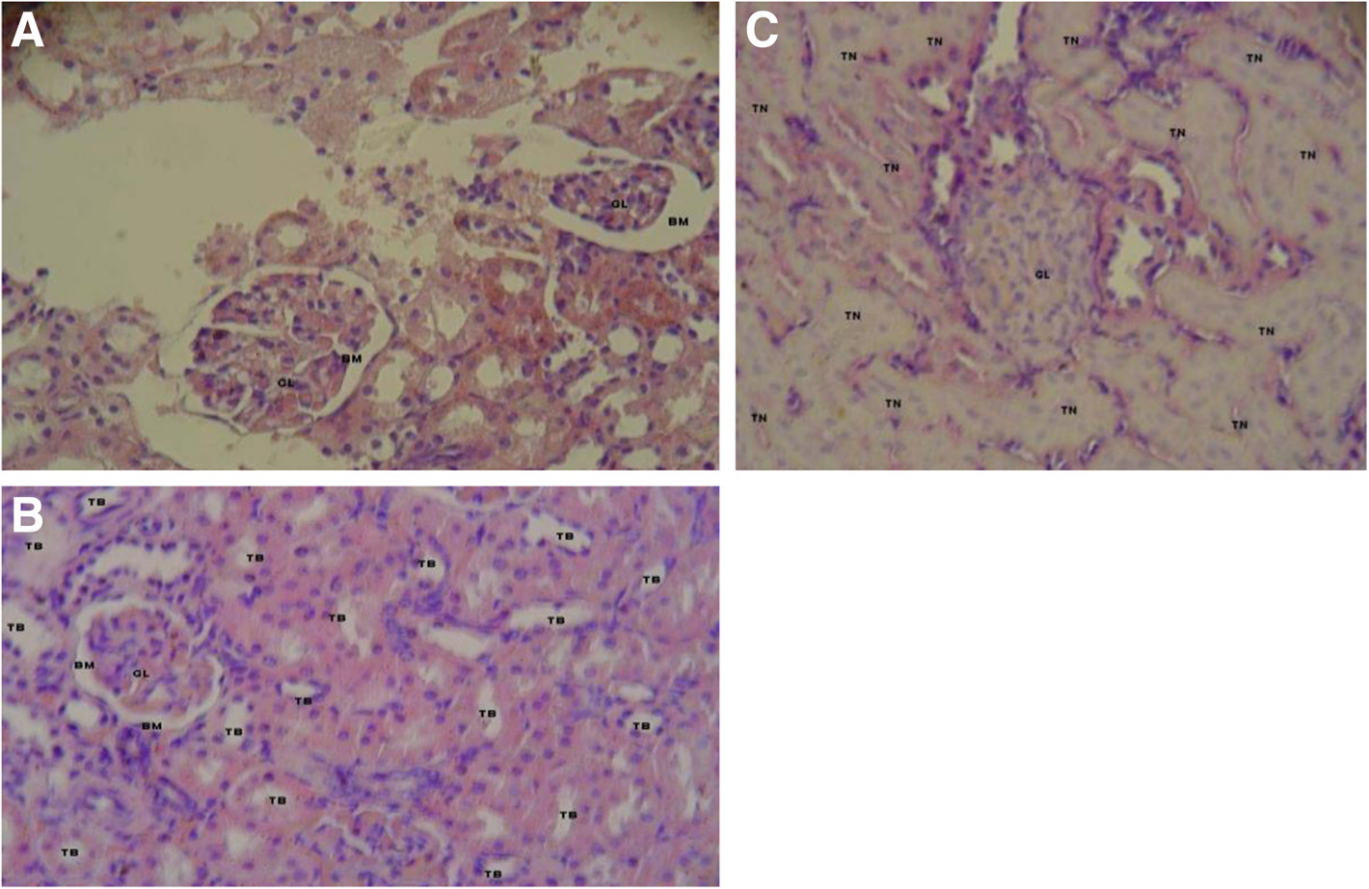
**A.: Histological section of kidney of control rat (H&E x400).** Section showing normal histological features including glomerulus and tubules. **B**: Histological section of test rat kidney at dose of 100 mg/kg (H&E x400). Section showing an intact glomerulus and tubules in extract-treated animals. **C**: Histological section of test rat kidney at dose of 1000 mg/kg (H& E x400). Section showing a glomerulus with loss of surrounding Bowman’s capsule, diffuse tubular necrosis in extract-treated animals at 1000 mg/kg.

There were no overt pathological lesions at lower dose levels studied. However, at doses ≥ 1000 mg/kg, histopathological changes including inflammation and necrosis were observed in both kidney and liver tissues (Figures 1 and 2).

## Discussion

Daily oral doses of the extract up to 100 mg/kg did not cause any obvious clinical abnormalities or death after eight weeks of treatment.

The estimation of some biochemical parameters such as the activities of enzymes in tissues and body fluids plays a major role in disease investigation, diagnosis and liver toxicity [28,29]. The biochemical parameters in the test group were to a large extent similar to the control at a dose of 100 mg/kg.

BUN values and ALT activity at a dose of 1000 mg/kg was significantly increased compared to the controls. The significant change in plasma ALT activity with insignificant change in plasma AST at the dose of 1000 mg/kg indicates that the extract caused mild changes in the liver. ALT is a cytoplasmic enzyme and increase in plasma is an indication of mild injuries caused by drugs to the liver. Liver injury is characterized as hepatocellular when there is predominant elevation of the ALT, while AST is a mitochondria enzyme whose increase activity in plasma reflects severe tissue injuries [30].

In the present study, the extract caused increase in ALT at a dose of 1000 mg/kg with AST and ALP activities falling within the normal limits at all dose levels. This indicates that the liver injury at a dose of 1000 mg/kg observed as reflected by an increase in ALT activity is hepatocellular and not cholestatic or intrabiliary in origin [31-33].

The observed dose- related significant increase in BUN values at a dose of 1000 mg/kg suggests that the extract at this oral dose level would cause renal damage. Hypo-proteinaemia a common finding in liver damage [29], was not observed in the present study, the total plasma proteins and albumin concentrations were not significantly affected at all dose levels. These findings indicate that the extract would not cause any overt liver damage at the dose levels studied. The non significant increase or decrease in the haematological values, is an indication that the extract does not affect the haemopoetic system when administered orally at the doses used in this study.

Furthermore, there were no discernable lesions at the dose up to 100 kg/mg. These findings further showed that the effect of the extract on target tissues namely liver and kidney as shown by biochemical data, is insignificant at the dose levels used. Therefore, the observed significant increase in plasma levels of ALT and BUN at 1000 mg/kg might have been caused by sub-cellular tissue changes.

It is plausible to infer that the ethanol extract of *Petroselinum crispum* is not toxic when administered orally at doses of 10-100 mg/kg for 8 weeks in rats. However, daily oral dose of 1000 mg/kg or greater can cause liver and kidney damage after 8 weeks of continued administration.

It is therefore expedient to advise that caution should be exercised in the use of Parsley in treatment of ailments to avoid overdosing bearing in mind the challenge of standardised dosing in herbal medicine due to paucity of information on pharmaco-genetics and pharmaco-dynamics of herbal preparations. In addition, consideration should be given to the potential toxicity of ingestion of Parsley with allopathic medication with serotonin activity. Furthermore, ingestion of parsley should be discouraged in pregnancy, lactating mothers and in individuals on opiods, lithium salts, diuretics and warfarin therapy due to potential drug-herb interaction [4,9,34]. Because Parsley bio-accumulate toxic heavy metals [12], it’s cultivation should not be done on soil irrigated with untreated waste water.

## Conclusion

These results indicate that the leaf ethanol extract of *Petroselinum crispum* was mildly hepatotoxic and nephrotoxic at continued oral doses equal to or more than 1000 mg/kg. It was concluded that the extract does not cause any obvious toxicity when used at lower doses, it is therefore expedient for users to exercise caution in its administration to avoid overdosing. In circumstances of possible contraindications like pregnancy, lithium ingestion as common in psychiatric patients, warfarin and opiods therapies, it’s use should be avoided. In addition, plant source should not be from soil irrigated with waste water.

## Competing interests

The authors hereby declared that there is no competing interest.

## Authors’ contributions

AEO: conceived of the study, was responsible for the conception of idea, design, plant collection and extraction and manuscript preparation. SOB: contributed to the writing of the manuscript, biochemical, haematological and histopathological analyses and interpretation. All authors read and approved the final manuscript.

## Pre-publication history

The pre-publication history for this paper can be accessed here:

http://www.biomedcentral.com/1472-6882/13/75/prepub
